# On cicadas of *Hyalessa maculaticollis* complex (Hemiptera, Cicadidae) of China

**DOI:** 10.3897/zookeys.369.6506

**Published:** 2014-01-13

**Authors:** Xu Wang, Masami Hayashi, Cong Wei

**Affiliations:** 1 Key Laboratory of Plant Protection Resources and Pest Management, Ministry of Education, Entomological Museum, Northwest A&F University, Yangling, Shaanxi 712100, China; 2 Department of Biology, Faculty of Education, Saitama University, Saitama 338-8570, Japan

**Keywords:** Cicadomorpha, *Oncotympana*, *Sonata*, variability, morphology, new combination

## Abstract

The genus *Hyalessa* China is reviewed based on the discovery of male of the type species *H. ronshana* China as well as the description of one new species (*H. batangensis* **sp. n.**). The species formerly included in the genus *Sonata* Lee are removed to *Hyalessa* as new combinations. Intraspecific variations of *H. maculaticollis* are enumerated based on materials collected from various locations from China. The identity of *Sonata* and the systematic placement of *Hyalessa* are discussed. A key to all species of *Hyalessa* is provided.

## Introduction

The *Oncotympana* was established by Stål in 1870 as a subgenus of *Pomponia* Stål, 1866. Distant (1905a) raised *Oncotympana* to generic level and redescribed it. Later, Ishihara (1961) established the tribe Oncotympanini to accommodate it, and Hayashi (1978, 1984) reviewed this genus. Chou et al. (1997) recorded four species of *Oncotympana* from China, but two of them (*Oncotympana stratoria* and *Oncotympana virescens*) were just listed in their checklist. Recently, Lee (2010) found that nine species of *Oncotympana* from the continental East Asia, Japan and India aren’t congeneric with the type species *Oncotympana pallidiventris*, so he established the genus *Sonata* to accommodate these species, i.e., *Sonata fuscata* (Distant), *Sonata maculaticollis* (Motschulsky), *Sonata ella* (Lei & Chou), *Sonata expansa* (Walker), *Sonata mahoni* (Distant), *Sonata melanoptera* (Distant), *Sonata obnubila* (Distant), *Sonata stratoria* (Distant), and *Sonata virescens* (Distant). More recently, Lee (2011) established the tribe Sonatini in the subfamily Cicadinae to contain the genus *Sonata*, and synonymised Oncotympanini with Cicadini and included the Philippine genera *Oncotympana* Stål and *Neoncotympana* Lee, 2011 in the subtribe Oncotympanina of Cicadini. However, Hayashi (2011) synonymised *Sonata* Lee with *Hyalessa* China, and transferred *Oncotympana maculaticollis* to the genus *Hyalessa*. Hayashi and Saisho (2011) recognized *Oncotympana fuscata* (Distant), the type species of *Sonata*, as a synonym of *Hyalessa maculaticollis* (Motschulsky).

In the present paper we review the genus *Hyalessa* based on the discovery of male of the type species *Hyalessa ronshana* China and the description of one new species, *Hyalessa batangensis* sp. n. from Southeast China. In addition, we transfer the species of *Sonata* to *Hyalessa*, bringing the species number of *Hyalyssa* to 10. Furthermore, the phenotypic variability of *Hyalessa maculaticollis*, the most widely distributed species among its congeners, is investigated based on materials collected from different locations.

## Materials and methods

This study is mainly based on specimens deposited in the following institutions abbreviated in the text as follows:

NWAFU Entomological Museum, Northwest A&F University, Yangling, China

BMNH The Natural History Museum, London, UK

MNHN Muséum National d’Histoire Naturelle, Paris, France

External morphology was observed using the Olympus SZX10 stereomicroscope, and photographed with a Nikon Coolpix P100 digital camera. The pygofer was carefully extracted from the terminal abdominal segments of relaxed specimen and observed and photographed using a Scientific Digital micrography system equipped with an Auto-montage imaging system and a QIMAGING Retiga 4000R digital camera (CCD). The extracted pygofer, if necessary, was dissected and placed in 10% KOH boiled for 2–5 minutes, washed, and transferred to glycerin for observation, and the aedeagus were photographed using CCD similarly. Terminology for morphological features follows that of Moulds (2005). All measurements are in millimeter.

The type specimens of the new species are deposited in the Entomological Museum, Northwest A&F University (NWAFU), Yangling, China.

## Systematics

### Family Cicadidae Latreille
Subfamily Cicadinae
Tribe Cicadini Latreille, 1802

#### Genus Hyalessa

China, 1925

http://species-id.net/wiki/Hyalessa

Pomponia (Oncotympana) Stål, 1870: 710. Type species: *Pomponia (Oncotympana) pallidiventris* Stål
Oncotympana : Distant 1905: 60, 70. Type species: *Pomponia (Oncotympana) pallidiventris* Stål
Sonata Lee, 2010: 20. Type species: *Oncotympana fuscata* Distant

##### Type species.

*Hyalessa ronshana* China.

Body small to large, head including eyes wider than pronotum. Anterolateral pronotal collar not dentate. Medial pronotal collar about one-fourth to one-third the length of inner area. Wings hyaline; fore wing with 8 apical cells, with fuscous spots at bases of apical cells second, third, fifth, and seventh, a marginal series of minute pale fuscous spots near apices of longitudinal veins to apical cells in most species; hind wing with 6 apical cells. Male operculum wider than long, lateral margin roundly produced laterad, overlapped or nearly touching to the other one centrally. Male abdomen slightly shorter than distance from head to cruciform elevation. Posterior margin of male abdominal tergite III much wider than mesonotum. Timbal concealed by timbal cover in dorsal view; timbal cover globolised, projecting beyond corresponding lateral margin of abdomen. Aedeagus thick and curved apically; apex with pair of sclerotized lateral processes and pair of (membranous) saccate hooks between them. Uncal lobe large, separated from the other one distally or connected to the other one from near base to subapex.

##### Remarks.

This genus is closely similar to *Oncotympana* Stål in habitus, but differs from the latter in the following characteristics: pronotum about or more than twice as long as head; anterolateral pronotal collar not dentate; male operculum shorter than wide but very large; uncal lobes bifurcated; aedeagus very thick, with apex with a pair of sclerotized lateral processes and a pair of (membranous) saccate hooks between them.

##### Key to the species of *Hyalessa*

**Table:** 

1	Fore wing with a faint spot merely at bases of apical cells second, third, fifth, and seventh, respectively	2
–	Fore wing with a distinct large fuscous spot at bases of apical cells second, third, fifth, and seventh and a marginal series of minute pale fuscous spots near apices of longitudinal veins to apical cells, respectively	3
2	Uncal lobes separated from the other one from middle of uncus, with posterior margin rounded and outer margin weakly convex	*Hyalessa ronshana*
–	Uncal lobes connected to each other closely from near base to apex, with lateral margin slightly concave basally and convex to distal margin	*Hyalessa batangensis* sp. n.
3	Male opercula well separated from each other, or close to each other but not overlapping	4
–	Male opercula overlapping medially	5
4	Male opercula entirely dark greenish ochraceous, and abdomen (both in males and females) with broad olivaceous or ochraceous markings	*Hyalessa mahoni* comb. n.
–	Male opercula entirely much infuscated to black	*Hyalessa melanoptera* comb. n.
5	Body smaller, body length < 26.0 mm	6
–	Body larger, body length > 30.0 mm	9
6	Male opercula not reaching hind margin of abdominal sternite II	*Hyalessa stratoria* comb. n.
–	Male opercula extending to hind margin of abdominal sternite II	7
7	Mesonotum green or ochraceous; opercula pale yellowish green	*Hyalessa virescens* comb. n.
–	Mesonotum black; opercula testaceous	8
8	Body smaller, body length about 13.0 mm; uncal lobes separated from each other from near base of uncus	*Hyalessa expansa* comb. n.
–	Body larger, body length about 25.0 mm; uncal lobes separated from each other from middle of uncus	*Hyalessa ella* comb. n.
9	Head narrower, about 0.73 times as wide as pronotum; uncal lobes broad, with outer margins convexly sinuate and inner margins nearly straight	*Hyalessa obnubila* comb. n.
–	Head broader, about 0.79 times as wide as pronotum; uncal lobes long, with inner margins parallel to outer margins	*Hyalessa maculaticollis*

#### Hyalessa ronshana

China

http://species-id.net/wiki/Hyalessa_ronshana

Figs 1 – 3

##### Material examined.

Holotype: ♀ (BMNH), China: Yunnan Prov., 31.VII.1922. 1♂ (NWAFU), China: Hutiaoxia, Xianggelila County, Yunnan Prov., 2.VII.2007, coll. Cong Wei; 10♂♂, 4♀♀ (NWAFU), China: Hutiaoxia, Xianggelila County, Yunnan Prov., 27.VII.2007, coll. Cong Wei; 7♂♂, 8♀♀ (NWAFU), China: Hutiaoxia, Xianggelila County, Yunnan Prov., 6.VIII.2010, coll. Meng Zhang; 1♀ (NWAFU), China: Hutiaoxia, Xianggelila County, Yunnan Prov., 6.VIII.2010, coll. Silong Xu; 1♂(NWAFU),China: Hutiaoxia, Xianggelila County, Yunnan Prov., 7.VIII.2010, coll. Meng Zhang.

##### Measurements of types.

(18 ♂♂, 12 ♀♀). Body length: male 31.3–37.8, female 32.6–35.7; fore wing length: male 45.6–50.3, female 46.6–53.3; fore wing width: male 15.2–17.6, female 15.4–17.5; width of head including eyes: male 11.0–12.0, female 10.4–12.0; pronotum width (including pronotal collar): male 14.0–16.0, female 14.0–16.2; mesonotum width: male 11.7–13.3, female 11.5–13.4.

##### Description of male.

Body almost black, with short yellow-green hairs.

Head (Fig. 2A, C) about 0.77 times as wide as pronotum; eyes fuscous, ocellus red. Postclypeus moderately swollen, black, with greenish transverse grooves on each side; lateral margin greenish. Anteclypeus black, with yellowish green medially. Rostrum long, extending to posterior trochanter.

Thorax (Fig. 2A, C). Pronotum generally black, with central longitudinal greenish yellow spot near anterior margin, smaller greenish yellow spot on disc, and central round greenish yellow spot near posterior margin; lateral margins of pronotal collar ampliate. Mesonotum black, with pair of greenish markings on anterior angles of cruciform elevation. Metanotum and lateral part of cruciform elevation yellowish green. Thoracic sternites greenish to black.

Legs (Fig. 2E). Black, fore femur with large ochraceous patch medially and smaller ochraceous patch near posterior margin in lateral view. Fore tibia and mid femur mostly black. Hind legs mostly ochraceous. Fore femur with primary spine longest and oblique to femur, secondary spine of intermediate size and subapical spine shortest, both angled slightly.

Wings (Fig. 2A–B). Hyaline, fore wing with distinct infuscation at bases of apical cells second, third, fifth, and seventh; a marginal series of minute pale fuscous spots near apices of longitudinal veins to apical cells.

Abdomen (Fig. 2A, D). Generally black dorsally, with white pollinosity between tergite II and III. Timbal cover black, prominently globolised. Opercula greyish green, centrally overlapping, with rounded posterior margin extending to abdominal sternite II. Abdominal sternites mostly black, with greenish speckle on sternite III, VII and VIII, sparsely covered with white pollinosity.

Genitalia (Fig. 2F, G, H). Pygofer barrel-shaped in ventral view. Uncal lobes broad and well developed, separated from the other one from middle of uncus, with posterior margin rounded and outer margin weakly convex. Basal lobe of uncus shorter, ca 1/2 length of uncal lobe. Aedeagus with apical one third strongly curved ventrally, expended subapically; sclerotized lateral processes acute, large medial (membranous) saccate hook somewhat truncate with a pair of small lateral membranous processes between sclerotized lateral processes.

##### Description of female.

**Figure 1.**
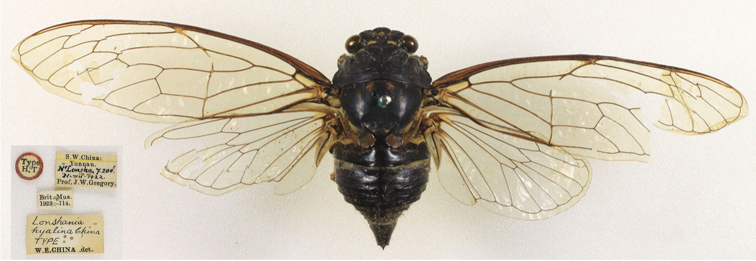
*Hyalessa ronshana* (female, holotype), habitus, dorsal view.

(Figs 1, 3). Opercula smaller than those of male, broadly separated from each other. Abdominal segment IX (pygofer) greenish; ovipositor sheath not extending beyond segment IX, posterior margin of segment VII incised at middle. Other characteristics similar to male.

**Figure 2.**
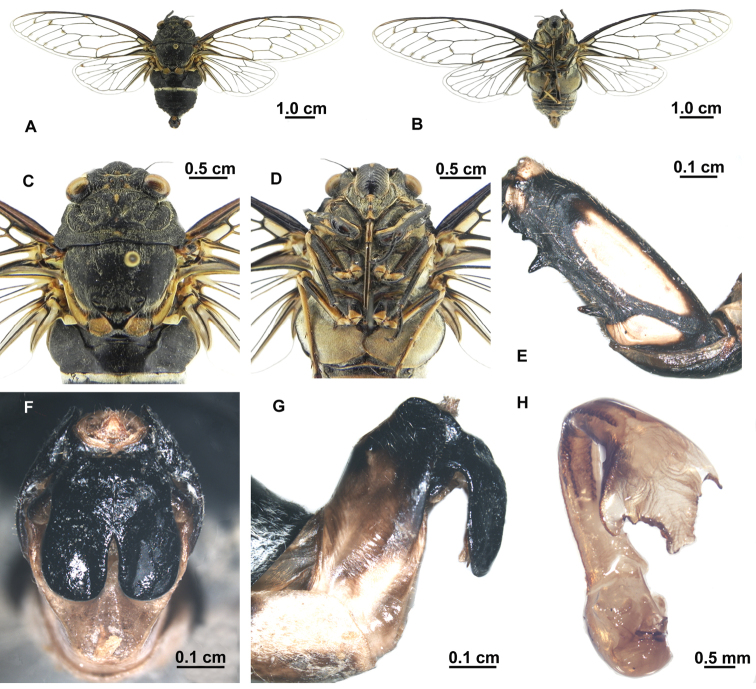
*Hyalessa ronshana* (male). **A** habitus, dorsal view **B** habitus, ventral view **C** head and thorax, dorsal view **D** head and thorax, ventral view **E** left foreleg, showing the spines on fore femur **F** male pygofer, ventral view **G** male pygofer, lateral view **H** aedeagus.

**Figure 3.**
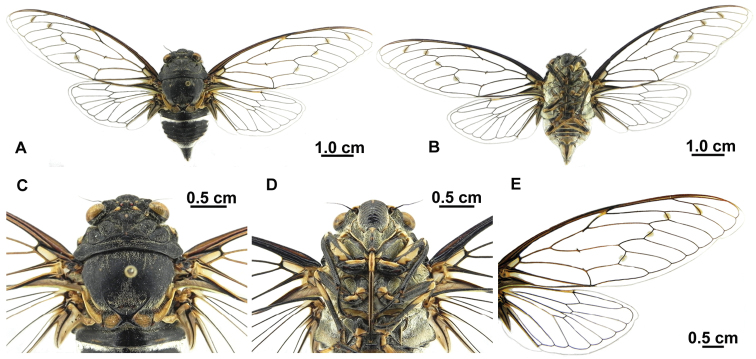
*Hyalessa ronshana* (female). **A** habitus, dorsal view **B** habitus, ventral view **C** head and thorax, dorsal view **D** head and thorax, ventral view **E** fore and hind wings.

##### Distribution.

China (Yunnan).

##### Remarks.

*Hyalessa* China formerly included only the type species *Hyalessa ronshana* which was established on a single female collected from Yunnan Prov., China. Recently, when we investigated materials of this genus collected from different locations from China, some specimens also from Yunnan Province were found very similar to *Hyalessa ronshana*, but they can be distinguished from the holotype of *Hyalessa ronshana* by the concoloured mesonotum (blackish, without paired large spots adjacent to the anterior margin of mesonotum), the normal nodal line of fore wing (absent in the ulnar cell 3 and the medial cell), the fuscous spots at bases of apical cells second, third, fifth, and seventh of fore wing, and a marginal series of minute pale fuscous spots near apices of longitudinal veins to apical cells. However, the holotype of *Hyalessa ronshana* is an unusual form, representing a kind of deformation on the forewing vein, i.e., the veins are somewhat asymmetric and particularly, the long nodal line presented on the ulnar cell 3 and the medial cell. In addition, the condition of the holotype is not good in condition, e.g., the faint markings on mesonotum are not strict, and only one very faint spot appeared at the base of apical cell second of fore wing (other infuscations on the veins of fore wing seem to be diminished due to the poor condition of the specimen). This is probably due to that the holotype was rather teneral and/or it has been deposited in the collection for a long time. Herein, judging from the adjacency of the forementioned materials collected from Yunnan Prov. and the holotype of *Hyalessa ronshana* as well as the common characters shared by them, particularly the transverse pollinosity-like band on base of abdominal tergite III, coloration of veins (both upperside and underside), maculation on fore femur, hind tibia, opercula and pygofer, we conclude that the new materials are conspecific with the holotype of *Hyalessa ronshana*, and redescribe this species based on the discovery of the male for the first time. *Hyalessa ronshana* is similar to *Hyalessa maculaticollis*, but can be distinguished from the latter by the generally black pronotum and mesonotum, the rounded apex of the broad uncal lobes of male pygofer, and the shape of apical hooks of aedeagus.

#### Hyalessa batangensis sp. n.

http://zoobank.org/F42CAD01-CECC-408F-A4EB-11B74EE23C34

http://species-id.net/wiki/Hyalessa_batangensis

Figs 4 –5

##### Type material.

Holotype: ♂ (NWAFU),China: Batang County, Sichuan Prov., 12.VIII.2001. Paratype: 1♂ (NWAFU),China: Batang County, Sichuan Prov., 12.VIII.2001.

##### Measurements of types.

(2♂♂): Body length: 26.6–31.8; fore wing length: 37.1–43.5; fore wing width: 12.4–15.2; width of head including eyes: 8.6–10.3; pronotum width (including pronotal collar): 11.3–14.0; mesonotum width: 9.4–10.9.

##### Etymology.

The species name is derived from the location of the types.

##### Description of male.

**Figure 4.**
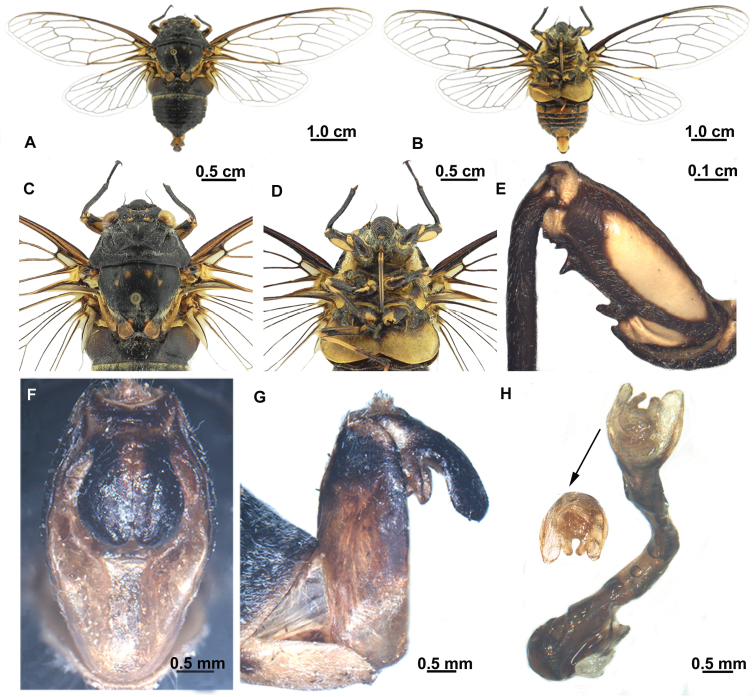
*Hyalessa batangensis* sp. n. (male, holotype). **A** habitus, dorsal view **B** habitus, ventral view **C** head and thorax, dorsal view **D** head and thorax, ventral view **E** left foreleg, showing the spines on fore femur **F** male pygofer, ventral view **G** male pygofer, lateral view **H** aedeagus.

Head (Fig. 4A, C) about 0.74 times as wide as pronotum. Compound eye greenish brown, ocellus red. Postclypeus moderately swollen, with black medial longitudinal fasciae and greenish yellow transverse grooves on each side. Anteclypeus black, with yellowish green fasciae medially. Rostrum with black apex extending to posterior trochanter.

Thorax (Fig. 4A, C). Pronotum and mesonotum almost black, pronotum with pair of submedian markings and pair of lateral markings yellow greenish. Mesonotum with pair of submedian markings and lateral markings respectively, or without distinct markings. Cruciform elevation black, with pair of yellow greenish markings on anterior angles. Metanotum and lateral part of cruciform elevation yellowish green. Thoracic sternites yellow greenish, with ochraceous patches.

Legs (Fig. 4E). Dark brown, fore femur with large yellowish ochraceous patch medially and smaller ochraceous patch near posterior margin in lateral view. Fore tibia and mid femur, tibia mostly dark brown. Fore femur with primary spine conical and less angled; secondary and subapical spines erect and pointed.

Wings (Fig. 4A, B). Hyaline, fore wing with indistinct infuscation at bases of apical cells second and third; no fuscous spots near apices of longitudinal veins to apical cells.

Abdomen (Fig. 4A, D). Black, with white pruinosity between tergite II and III. Timbal cover brownish ochraceous, circular and globose. Opercula yellow greenish, centrally overlapping, with rounded apex extending to posterior margin of sternite II. Abdominal sternites mostly black, with sternite VII, VIII and posterior margin of III, IV, V and VI yellow greenish.

Genitalia (Fig. 4F, G, H). Pygofer barrel-shaped in ventral view. Uncal lobes connected to each other closely from near base to apex; lateral margin of uncal lobe slightly concave basally and convex to distal margin. Aedeagus with apex curved ventrally, expended subapically; apex with sclerotized lateral processes very broad and rounded, pair of (membraneous) saccate hooks between sclerotized lateral processes.

##### Female.

Unknown.

##### Distribution.

China (Sichuan).

##### Remarks.

**Figure 5.**
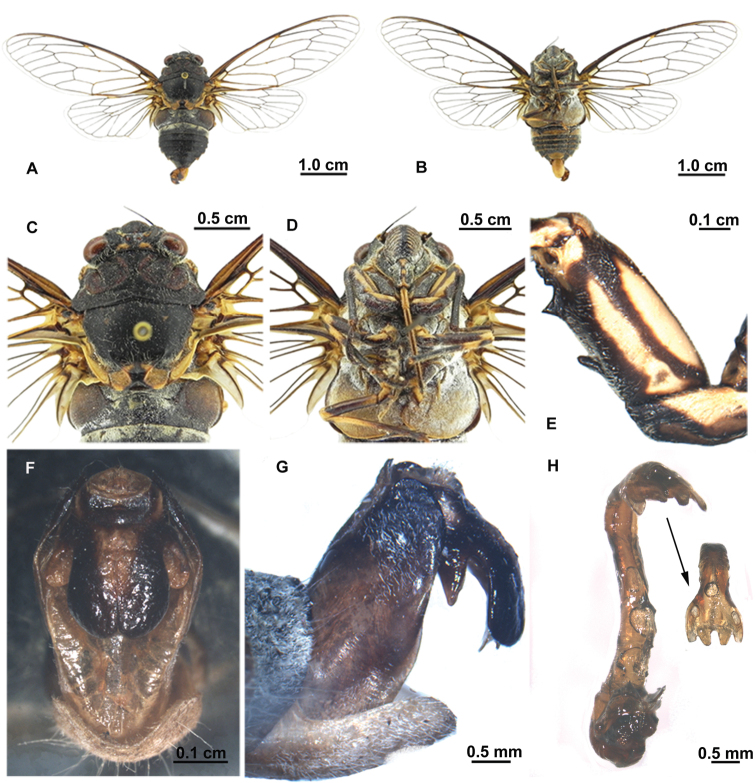
*Hyalessa batangensis* sp. n. (male, paratype). **A**. habitus. dorsal view **B** habitus, ventral view **C** head and thorax, dorsal view **D** head and thorax, ventral view **E** left foreleg, showing the spines on fore femur **F** male pygofer, ventral view **G** male pygofer, lateral view **H** aedeagus.

This new species can be distinguished from its congeners by the combination of the following characters: slender body, without fuscations on veins of fore wing, and uncal lobes connected to each other closely from near base to apex. There are slight differences of body size, markings on mesonotum, and the shape of aedeagus presents between the holotype and the paratype: the holotype has a bigger body size (31.8 mm), a pair of submedian markings and a pair of lateral markings on mesonotum, and a pair of broad, rounded sclerotized lateral processes on aedeagus (Fig. 4); the paratype has a smaller body size (26.6 mm), without distinct markings on mesonotum, and the sclerotized lateral processes on aedeagus are short and acute (Fig. 5). We tentatively treat the latter as an intraspecific variation of this species, and its identity needs to be confirmed when more materials are available.

#### Hyalessa maculaticollis

(Motschulsky)

http://species-id.net/wiki/Hyalessa_maculaticollis

Cicada maculaticollis Motschulsky, 1866: 185.
Pomponia maculaticollis Distant, 1888: 296.
Oncotympana maculaticollis : Distant 1905: 559.
Oncotympana fuscata Distant, 1905: 558.
Oncotympana coreanus Kato, 1925: 27.
Sonata maculaticollis Lee, 2010: 20.

##### Material examined.

1♂ (NWAFU), China: Mt. Emei, Sichuan Prov., 5.V.1957; 1♂ (NWAFU), China: Mt. Emei, Sichuan Prov., 22.VII.1974; 8♂♂ (NWAFU), China: Mt. Emei, Sichuan Prov., 22.VII.1991; 4♂♂ (NWAFU), China: Mt. Emei, Sichuan Prov., 26.VII.1991; 1♂ (NWAFU),China: Mt. Emei, Sichuan Prov., 23.VII.2009; 1♂ (NWAFU), China: Beijing, 14.VII.1961; 1♂ (NWAFU), China: Beijing, 18.VII.1961; 3♂♂ (NWAFU), China: Qingdao, Shandong Prov., 29.VII.2010; 3♂♂ (NWAFU), China: Qingdao, Shandong Prov., 2.VIII.2010; 3♂♂ (NWAFU), China: Qingdao, Shandong Prov., 4.VIII.2010; 5♂♂ (NWAFU), China: Qingdao, Shandong Prov., 8.VIII.2010; 1♂ (NWAFU), China: Chunhua County, Shaanxi Prov., 6.VIII.1981; 1♂ (NWAFU), China: Guangdong Prov., 5.VIII.1957; 2♂♂ (NWAFU), China: Mts. Shennongjia, Hubei Prov., 11.VIII.2004; 1♂ (NWAFU), China: Mts. Shennongjia, Hubei Prov., 15.VIII.2004; 2♂♂ (NWAFU), China: Huoditang, Ningshan County, Shaanxi Prov., 5.VIII.2008; 2♂♂ (NWAFU), China: Mt. Nanwutai, Xi’an, Shaanxi Prov., 25.VII.1951; 2♂♂ (NWAFU), China: Mt. Nanwutai, Xi’an, Shaanxi Prov., 26.VIII.1957; 1♂ (NWAFU), China: Mt. Nanwutai, Xi’an, Shaanxi Prov., 13.VII.1959; 1♂ (NWAFU), China: Mt. Tianmu, Zhejiang Prov., 15.VIII.1965; 1♂ (NWAFU), China: Mt. Tianmu, Zhejiang Prov., 26.VII.2003; 2♂♂ (NWAFU), China: Mt. Tianmu, Zhejiang Prov., 28.VII.2003; 1♂ (NWAFU), China: Mt. Tianmu, Zhejiang Prov., 29.VII.2003; 31♂♂ (NWAFU), China: Mt. Huping, Hunan Prov., 26.VII.2013; 4♂♂ (NWAFU), China: Mt. Qingcheng, Sichuan Prov., 9.VIII.2013.

##### Main characters.

Body large, head slightly shorter than base of mesonotum in dorsal view. Rostrum extending to the posterior trochanter. Mesonotum black with following green markings: two large central obconical spots, three pairs of large greenish spots around them and pair of greenish spots on each lateral margin. Abdomen black; timbal cover ochraceous. Wings hyaline; fore wing with large fuscous spot at bases of apical cells second, third, fifth, and seventh; a marginal series of minute pale fuscous spots near apices of longitudinal veins to apical cells. Opercula in male broad, convex, extending to posterior margin of second abdominal segment, and overlapping; opercula in female smaller than those of male and broadly separated from each other. Aedeagus thick and curve ventrally, expended subapically, with a pair of sclerotized lateral processes apically as well as a pair of (membraneous) saccate hooks when everted.

##### Remarks.

After examining the holotype (male) of *Oncotympana fuscata* Distantpreserved in the Muséum National d’Histoire Naturelle (by MH) and investigating the intraspecific variability of *Hyalessa maculaticollis*, we reconfirm that *Hyalessa fuscata* is a junior synonym of *Hyalessa maculaticollis*, as previously proposed by several authors. Among the species of *Hyalessa*, *Hyalessa maculaticollis* has the widest range of distribution, from the Russian Maritime Territory, Korean Peninsula, Japan to China. This species mainly occurs in the forests, and the calling song of males is very loud with a complex transposition. *Hyalessa maculaticollis* is noted for its great intraspecific variability, including body size, markings on thorax, timbal cover, opercula and aedeagus, which has been recorded by Hayashi and Saisho (2011) based on materials collected from Japan. In this study, based on more materials collected from different locations in China, we further investigate the intraspecific variability of this species. For details, see below.

##### Intraspecific variability.

**Figure 6.**
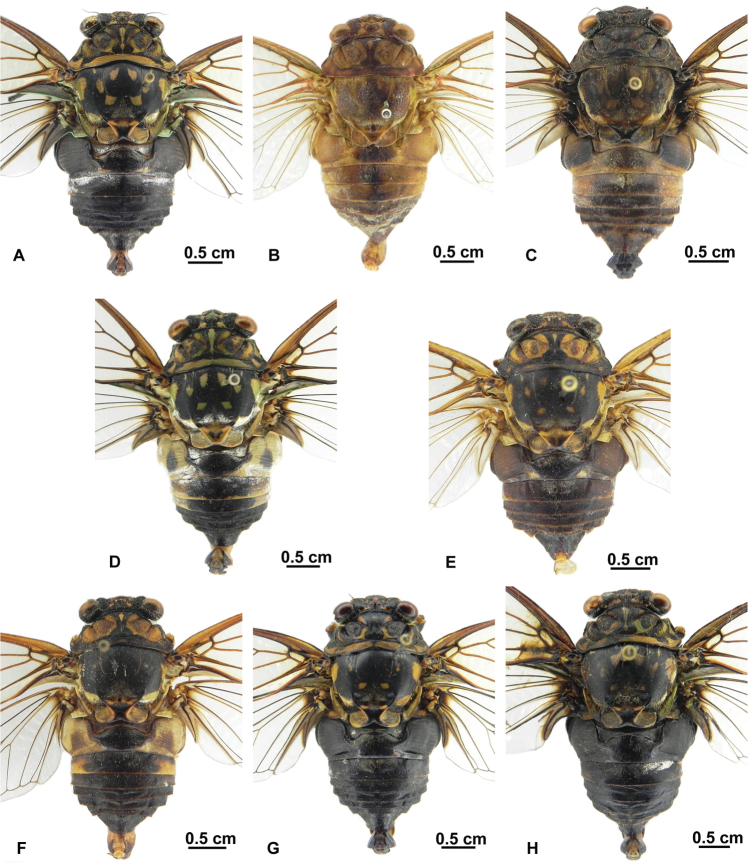
*Hyalessa maculaticollis* (male). Habitus, dorsal view, showing intraspecific variability in Chinese populations. **A** material from Mt. Emei, Sichuan Prov. **B** material from Beijing **C** material from Qingdao, Shandong Prov. **D** material from Qingdao, Shandong Prov. **E** material from Chunhua County, Shaanxi Prov. **F** material from Guangdong Prov. **G** material from Mts. Shennongjia, Hubei Prov. **H** material from Huoditang, Ningshan County, Shaanxi Prov.

**Body size.** (1) Medium, about 30 mm in length (Fig. 6F); (2) Large, about 36 mm in length (Fig. 6A).

**Coloration of body.** (1) Generally black with green or ochreous markings (Fig. 6A, D, G, H); (2) Generally yellowish with dark ochreous markings (Fig. 6B); (3) Variegated (Fig. 6C, E, F).

**Markings on mesonotum.** (1) Mesonotum with 5 pairs of greenish spots: a pair of very small ones near anterior margin, three large spots on disc, and a pair of very lagre spots on lateral margins, (Fig. 6A, D); (2) Above mentioned 5 pairs of spots on mesonotum obscure or reduced (Fig. 6C, E, G, H); (3) Mesonotum with no distinct markings on disc (Fig. 6B, F).

**Timbal cover.** (1) Black (Fig. 6A, G, H); (2) Ochreous or greenish yellow, with dark ochreous to black patch on posterior area (Fig. 6C, D, F); (3) Dark ochreous or yellow (Fig. 6B, E).

**Figure 7.**
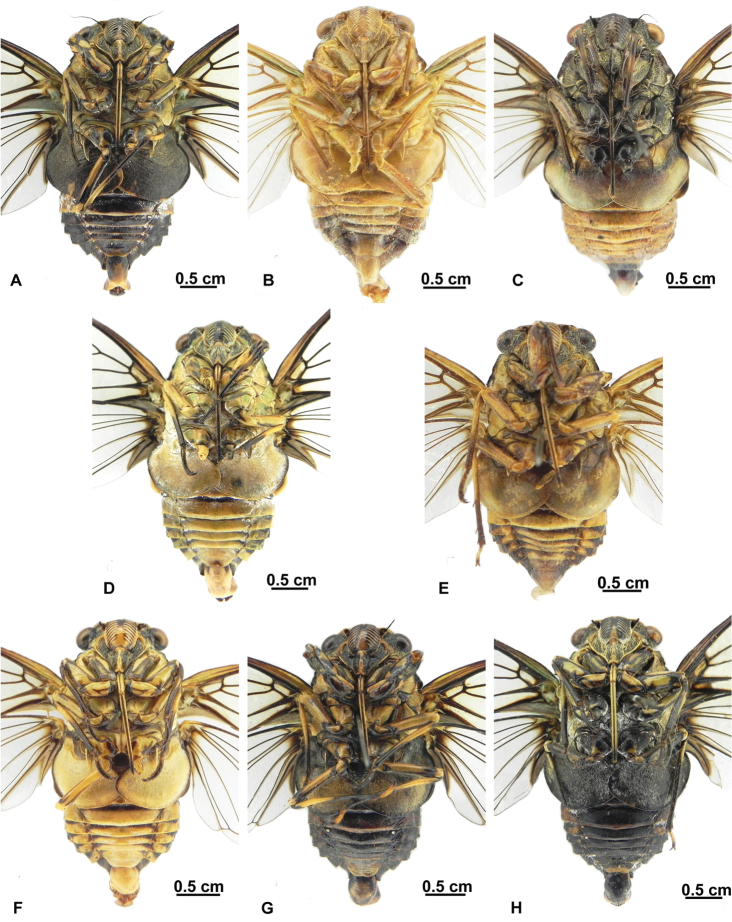
*Hyalessa maculaticollis* (male). Habitus, ventral view, showing intraspecific variability in Chinese populations. **A** material from Mt. Emei, Sichuan Prov. **B** material from Beijing **C** material from Qingdao, Shandong Prov. **D** material from Qingdao, Shandong Prov. **E** material from Chunhua County, Shaanxi Prov. **F** material from Guangdong Prov. **G** material from Mts. Shennongjia, Hubei Prov. **H** material from Huoditang, Ningshan County, Shaanxi Prov.

**Male opercula.** (1) Black (Fig. 7A, H); (2) Light ochreous or brown (Fig. 7B, F); (3) Variegated (Fig. 7C, D, E, G).

**Figure 8.**
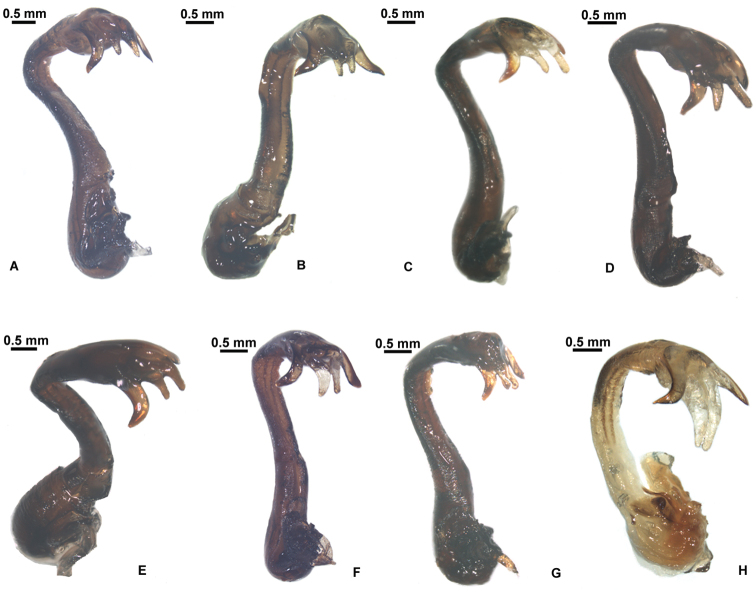
*Hyalessa maculaticollis* (male). Aedeagus, lateral view, showing intraspecific phenotypic variability in Chinese populations. **A** material from Qingdao, Shandong Prov. **B** material from Mt. Tianmu, Zhejiang Prov. **C** material from Huoditang, Ningshan County, Shaanxi Prov. **D** material from Mt. Qingcheng, Sichuan Prov. **E** material from Mt. Huping, Hunan Prov. **F** material from Mts. Shennongjia, Hubei Prov. **G–H** material from Mt. Nanwutai, Xi’an, Shaanxi Prov.

**Shaft of aedeagus.** (1) Aedeagal shaft slightly S-shaped in lateral view (Fig. 8A, B, C, D, F, G); (2) Aedeagal shaft S-shaped in lateral view (Fig. 8E); (3) Aedeagal shaft C-shaped in lateral view (Fig. 8H).

**Apex of aedeagus.** (1) Paired saccate hooks much shorter than the sclerotized lateral processes (Fig. 8A, B, E); (2) Paired saccate hooks slightly shorter than the sclerotized lateral processes (Fig. 8C, D, F, G); (3) Paired saccate hooks much longer than the sclerotized lateral processes (Fig. 8H).

## Discussion

The genus *Hyalessa* formerly included only the type species *Hyalessa ronshana* which was established on a single female collected from Yunnan Province of China. Herein, we re-address the identity of this species based on the discovery of the male for the first time and treat *Sonata* as a junior synonym of *Hyalessa*. This genus is retained in the tribe Cicadini, and its tribal and sub-tribal status awaiting further phylogenetic studies.

Regarding the validity of *Oncotympana fuscata*, Distant (1905) stated that *Oncotympana fuscata* is allied to *Oncotympana maculaticollis* but can be distinguished from the latter by the coloration of body, the narrower fore wings and the broader cruciform elevation. Kurosawa (1969) treated the populations in Korea and Far East Russia as a subspecies, *Oncotympana maculaticollis fuscata*. However, *Oncotympana fuscata* was treated as a junior synonym of *Oncotympana maculaticollis* by Chou et al. (1997). Lee (1999) applied the name *Oncotympana fuscata* to the Korean population because of substantial differences in song. Recently, Lee (2008) also treated *Oncotympana fuscata* and *Oncotympana maculaticollis* as two separated species and synonymized *Oncotympana maculaticollis fuscata* with *Oncotympana fuscata*. More recently,Lee (2010) proposed the genus *Sonata* with *Oncotympana fuscata* as its type species. However, Hayashi and Saisho (2011) synonymised *Sonata* with *Hyalessa* and treated *Hyalessa fuscata* as a junior synonym of *Hyalessa maculaticollis* based on examination of related holotypes deposited in the Muséum National d’Histoire Naturelle.

According to Hayashi and Saisho (2011), *Hyalessa maculaticollis* shows a high degree of variability in coloration. Based on more materials collected from China, we investigate further the variability of *Hyalessa maculaticollis* in this study, and the results show that greater intraspecific variations occur in this species, with respect to body size, markings on thorax, coloration of timbal cover and opercula and, in particular, the morphology of aedeagus that has never been described in detail for this species and its allies. Remarkably, the aedeagus of *Hyalessa maculaticollis* is furnished with a pair of apical sclerotized processes as well as a pair of saccate hooks. The relative lengths of the paired saccate hooks and the sclerotized lateral processes on aedeagus may be variable due to the scalability of the saccate hooks, which forms a continuous variation as a cline. Furthermore, the condition of curvature at apical 1/3 of aedeagus is also variable, particularly the aedeagal shaft of specimen collected from Mt. Nanwutai of Xi’an, Shaanxi Province (Fig. 8H) is C-shaped in lateral view, which is unique and can be easily distinguished from others with aedeagal shaft S-shaped (Fig. 8A–G). We tentatively treat this specimen as a variation of *Hyalessa maculaticollis*, but it may represent a new species of *Hyalessa*, which merits further studies using multiple sources such as morphology, acoustics, biology and molecular data, *etc*.

## Supplementary Material

XML Treatment for Hyalessa

XML Treatment for Hyalessa ronshana

XML Treatment for Hyalessa batangensis

XML Treatment for Hyalessa maculaticollis
